# Massive intraventricular hemorrhage caused by giant intracranial aneurysms

**DOI:** 10.11604/pamj.2021.39.262.29187

**Published:** 2021-08-24

**Authors:** Hassan Baallal, Ali Akhaddar

**Affiliations:** 1 Department of Neurosurgery, Avicenne Military Teaching Hospital, University Caddi Ayyad, Marrakech, Morocco

**Keywords:** Intraventricular, hemorrhage, giant, intracranial aneurysms

## Image in medicine

A 76-year-old man presented to the emergency department with a 2-month history of progressive headache, nausea, vomiting, irritability, and left focal seizures beginning as paresthesia´s and tonic clonic movements of the left lower limb subsequently spreading to the left upper limb. His medical history was notable for untreated hypertension and hyperlipidemia. He had been an active smoker for more than 20 years, and his father had died suddenly at 62 years of age from an unknown cause. There was no fever and no weight loss. At the time of admission her glasgow coma score (GCS) was 15 and there was no focal neurological deficit. The patient´s blood pressure was 160/80mmHg. Three hours later a brutal neurological fall-down was noticed after with a severe left sided hemiparesis. His blood test results, including complete blood count, erythrocyte sedimentation rate (ESR), and C-reactive protein (CRP) level were in the normal range. Brain computed tomography scan discovered a bilobed and bihemispheric huge mass (60 and 52 mm in diameter) of both frontal lobes with diffuse intraventricular hemorrhage necessitating emergent placement of an external ventricular drain, and the patient was admitted to the intermediate care unit.

**Figure 1:**
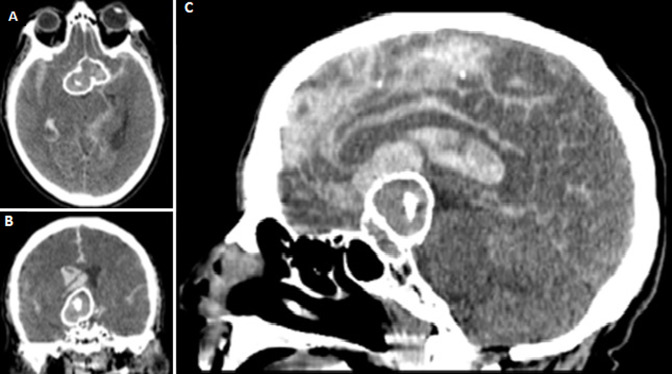
(A,B,C) brain computed tomography scan discovered a bilobed and bihemispheric huge mass (60 and 52 mm in diameter) of both frontal lobes with diffuse intraventricular hemorrhage

